# Biocatalytic activity of *Monascus* mycelia depending on physiology and high sensitivity to product concentration

**DOI:** 10.1186/s13568-017-0391-4

**Published:** 2017-04-27

**Authors:** Fengling Lu, Yaolin Huang, Xuehong Zhang, Zhilong Wang

**Affiliations:** 1 School of Pharmacy, State Key Laboratory of Microbial Metabolism, and Engineering Research Center of Cell & Therapeutic Antibody, Ministry of Education, Shanghai Jiao Tong University, Shanghai, 200240 People’s Republic of China; 2 School of Life Science and Biotechnology, and State Key Laboratory of Microbial Metabolism, Shanghai Jiao Tong University, Shanghai, 200240 People’s Republic of China

**Keywords:** Whole cell biocatalyst, Cultivation condition, Microbial physiology, Product degradation/inhibition

## Abstract

Cell suspension culture using mycelia as whole cell biocatalyst for production of orange *Monascus* pigments has been carried out successfully in a nonionic surfactant micelle aqueous solution. Thus, selection of mycelia as whole cell biocatalyst and the corresponding enzymatic kinetics for production of orange *Monascus* pigments can be optimized independently. Mycelia selected from submerged culture in a nonionic surfactant micelle aqueous solution with low pH 2.5 exhibits robust bioactivity. At the same time, enzymatic kinetic study shows that the bioactivity of mycelia as whole cell biocatalyst is sensitive to high product concentration. Segregation of product from mycelia by cell suspension culture in a nonionic surfactant micelle aqueous solution or peanut oil–water two-phase system is not only necessary for studying the enzymatic kinetics but also beneficial to industrial application of mycelia as whole cell biocatalyst.

## Introduction

Whole cell biocatalyst has been applied for production of valuable compound involving multi-step enzymatic biosynthesis as well as bio-redox reaction involving cofactor regeneration. In the case that product formation and cell growth are coupled, such as production of intracellular products, microbial fermentation using growing cells is applied, in which process optimization aiming at maximizing product usually defines biomass as objective function (Chen et al. 2015). On the contrary, when product formation is decoupled from cell growth, cell suspension culture using resting cells, i.e., the cells in no-growth or limited growth state by depletion of a compulsory nutrient element, is preferable. Cell suspension culture using resting cells exhibits some advantages in comparison with microbial fermentation using growing cells. Firstly, non-sterilization operation is possible due to the fact that the resting cell operation mode is carried out under nutrient limited condition (Sun et al. 2015). Secondly, high cell density is usually adopted during cell suspension culture, with which high product productivity can be achieved (Tsuge et al. 2014). Thirdly, high product yield based on energy source is also possible due to the avoidance of energy for biomass formation (Julsing et al. 2012). Furthermore, selection of whole cells as robust biocatalyst and optimization of enzymatic kinetics of whole cell biocatalyst can be carried out independently due to the decoupling of product formation from cell growth (Wang et al. 2005; Willrodt et al. 2016).

The bioactivity of whole cell biocatalyst is related to microbial physiology (Cornelissen et al. 2011). The microbial physiology is related to cultivation condition. There are many reports on the effect of cultivation conditions on the bioactivity of whole cell biocatalyst (Chen et al. 2011; Olaofe et al. 2013; Ramesh et al. 2016). On the other hand, there are few reports on the effect of mycelia culture period on bioactivity of whole cell biocatalyst (Mascotti et al. 2012). Whole cells utilized as biocatalyst are usually harvested at the late logarithm phase during growing cell submerged culture (Oremland et al. 1999). In addition, even robust biocatalyst may exhibit very low bioactivity under an unfavorable enzymatic kinetic condition. For example, product inhibition (Julsing et al. 2012; Kuhn et al. 2013) as well as product degradation (Winter et al. 2014) usually leads to low apparent bioactivity. Segregation of product from the whole cell biocatalyst is an efficient strategy for prevention of product inhibition on the biocatalyst or elimination of product degradation, which can be achieved by process engineering. In-situ product removal, such as addition of solid-state adsorbent (Evanst and Wang 1984), nonaqueous two-phase extraction (Wang and Dai 2010), is the most common strategy for elimination of product inhibition/degradation. Cascade conversion of instable/toxic product into stable/non-toxic compound by enzymatic reaction (Willrodt et al. 2015) or non-enzymatic reaction (Xiong et al. 2015; Domaille et al. 2016; Wallace and Balskus 2016) is also developed for bioprocess optimization.

*Monascus* is an ascomycete fungi widely utilized as a microbial source for production of natural pigments, including three major groups of *Monascus* pigments (red ones, monascorubramine and rubropunctamine; yellow ones, monascin and ankaflavin; and orange ones, rubropunctatin and monascorubrin) (Feng et al. 2012). The pigment profile as well as other secondary metabolites is strongly affected by the cultivation condition. Microbial fermentation at low pH leads to the accumulation of intracellular orange *Monascus* pigments (Kang et al. 2013), inhibition of citrinin production (Kang et al. 2014), and high lipid content in biomass (Wang et al. 2015). On the contrary, nearly neutral pH usually leads to accumulation of extracellular red *Monascus* pigments, production of toxic citrinin, and very low lipid content. Furthermore, nitrogen source in the cultivation medium strongly affects the pH of fermentation medium during fermentation process without pH control. Thus, nitrogen sources in the defined media (Kang et al. 2013, 2014) as well as complex media (Xiong et al. 2014) also influences the profile of *Monascus* pigments and citrinin accumulation. The influence of nitrogen concentration on profiling of *Monascus* proteome is also reported (Lin et al. 2008).

Submerged culture of *Monascus* sp. usually accumulates intracellular orange *Monascus* pigments under low pH condition. Microbial fermentation with high cell density can be applied for intensified production of intracellular *Monascus* pigments (Chen et al. 2015). In our previous work, extractive fermentation in a nonionic surfactant micelle aqueous solution has successfully released the intracellular *Monascus* pigments into its extracellular broth, meanwhile mycelia with very limited of intracellular *Monascus* pigments are achieved (Hu et al. 2012). Utilizing those mycelia as whole cell biocatalyst, cell suspension culture in a nonionic surfactant micelle aqueous solution (Wang et al. 2016) as well as plant oil–water two-phase system (Hu et al. 2016) is also carried out successfully. Thus, selection of mycelia as whole cell biocatalyst and optimization of enzymatic kinetics during biocatalytic process can be carried out independently.

In the present work, the relationship between cultivation condition, microbial physiology, and bioactivity of mycelia as whole cell biocatalyst for production of orange *Monascus* pigments was studied systemically. Firstly, the influence of cultivation conditions, such as culture period, carbon/nitrogen concentration, and pH, on microbial physiology, such as lipid content in biomass and intracellular *Monascus* pigment concentration, was examined. Then, the bioactivity of various mycelia as whole cell biocatalyst for production of orange *Monascus* pigments was determined in a nonionic surfactant micelle aqueous solution. At the same time, the corresponding enzymatic kinetic was further checked in a nonionic surfactant micelle aqueous solution and a plant oil–water two-phase system, respectively.

## Materials and methods

### Strain and culture media

*Monascus anka* (China Center of Industrial Culture Collection, CICC 5013) was used in this study. The strain was maintained on potato dextrose agar (PDA) medium (potato 200 g, glucose 20 g, and agar 15–20 g, per liter of tap water) at 4 °C.

The seed culture medium consisted of glucose 20 g, (NH_4_)_2_SO_4_ 4 g, peptone 10 g, KCl 0.5 g, KH_2_PO_4_ 4 g, and FeSO_4_∙7H_2_O 0.01 g, per liter of tap water. Inoculum culture was carried out in a 250 ml Erlenmeyer flask with working volume 50 ml at 30 °C and 200 rpm for 30 h.

### Collection of mycelia as whole cell biocatalyst

Submerged culture of *Monascus* sp. was carried out in a fermentation medium in the presence of nonionic surfactant (Hu et al. 2012). The fermentation medium consisted of nonionic surfactant Triton X-100 70 g, KH_2_PO_4_ 2.4 g, K_2_HPO_4_ 2.4 g, FeSO_4_∙7H_2_O 0.01 g, and ZnSO_4_∙7H_2_O 0.01 g, per liter of tap water while glucose, monosodium glutamate (MSG), as well as the initial pH [adjustment with 10% (V/V) hydrochloric acid] were listed in Table 1. After inoculum culture, 2 ml of inoculum culture were added into 250 ml Erlenmeyer flask with 50 ml of every entry of fermentation medium (Table 1), which was incubated at 30 °C and run 200 rpm. At a specified culture period, such as the 6th day, mycelia in the culture medium were collected by filtration and rinsed with pH 2 water (50 ml) to scour off the residual nutrients as well as nonionic surfactants. Dry cell weight (DCW), lipid content, and the concentration of intracellular *Monascus* pigments in mycelia were determined.

**Table 1:** Collection of mycelia from various cultivation conditions

Entry	Glucose (g/l)	MSG (g/l)	pH
1	*20*	*1.5*	*4.5*
2	*20*	*15*	*4.5*
3	50	5	4.5
4	50	5	2.5
5	*35*	*5*	*2.5*
6	*20*	*5*	*2.5*

Italics, underline, and italicunderline values are represented the influence of MSG concentration, pH, and glucose concentration, respectively

### Determination of mycelia bioactivity

The bioactivity of mycelia as whole cell biocatalyst was determined by cell suspension culture in a nonionic surfactant micelle aqueous solution. The nonionic surfactant micelle aqueous solution consisted of nonionic surfactant Triton X-100 70 g, glucose 50 g, KH_2_PO_4_ 2.4 g, K_2_HPO_4_ 2.4 g, FeSO_4_∙7H_2_O 0.01 g, and ZnSO_4_∙7H_2_O 0.01 g, per liter of tap water with an initial pH 5.5. A certain amount of wet mycelia (1.8 g wet mycelia, corresponding to approximately 10 g lipid-free DCW per liter) was added into 25 ml of the nonionic surfactant micelle aqueous solution in a 100 ml flask. The cell suspension aqueous solution was incubated at 30 °C and run 200 rpm for 42 h. The final pH reached to approximately 3.5. Then, DCW, lipid content, intracellular *Monascus* pigment concentration, and extracellular one, were determined.

### Time course of cell suspension culture

Time course of cell suspension culture using mycelia as whole cell biocatalyst was carried out in a nonionic surfactant micelle aqueous solution and peanut oil–water two-phase system, respectively. Cell suspension culture in a nonionic surfactant micelle aqueous solution was conducted under the same condition as the determination of mycelia bioactivity. For cell suspension culture in peanut oil–water two-phase system, Triton X-100 was replaced by peanut oil as extractant with volume ratio of peanut oil to aqueous solution equaling 1:1. The above micelle aqueous solution (25 ml) or oil–water two-phase system (25 ml aqueous phase) containing mycelia (1.8 g wet mycelia, corresponding to approximately 10 g lipid-free DCW per liter) was filled into 100 ml flask. A series of flasks were incubated at 30 °C and run 200 rpm under the same condition. At a certain time interval, three flasks were fetched for analysis of DCW, lipid content, intracellular *Monascus* pigment concentration, and extracellular ones.

### Analysis methods

DCW and lipid content were determined as detailed in our previous work (Wang et al. 2015). Mycelia after filtration from the culture broth were washed three times with equal volume of water [adjustment of pH to 2 with 10% (V/V) hydrochloric acid]. Wet mycelia were dried at 110 °C for at least overnight until constant mycelia weight reached. Datum of DCW was determined by gravity. Lipid weight in the dry mycelia was determined following the standard method (Bligh and Dyer 1959). For lipids might also be considered as a kind of secondary metabolite (Ratledge 2004), biomass was represented by lipid-free DCW (deduction of lipid weight from DCW) and lipid content was represented by ratio of lipid weight to lipid-free DCW.

Intracellular *Monascus* pigment concentration was determined as detailed in our previous work (Kang et al. 2013). Briefly, qualitative wet mycelia (such as 0.3 g) were incubated in 10 ml ethanol aqueous solution (70%, V/V, pH 2) at 30 °C and run at 200 rpm for 1 h. The intracellular *Monascus* pigments had been extracted completely into the ethanol aqueous solution. The absorbance of *Monascus* pigments in the ethanol aqueous solution was determined at 470 nm and represented as absorbance unit (AU, multiplication of the absorbance with its dilution ratio for a certain sample). Based on the datum of the corresponding lipid-free DCW, the concentration of intracellular *Monascus* pigments was achieved. There were small differences among the initial biomass loading in every entry of cell suspension culture. In order to eliminate the influence of those small differences in every entry, the concentrations of intracellular *Monascus* pigments before and after cell suspension culture were normalized to initial biomass concentration of 10 g lipid-free DCW per liter.

After removal of mycelia from the culture broth by filtration, the supernatant of filtrate was used to analyze extracellular *Monascus* pigment concentration. The supernatant (1 ml) was diluted properly with ethanol aqueous solution (70%, V/V, pH 2). The absorbance of *Monascus* pigments in the ethanol aqueous solution was determined at 470 nm and represented as absorbance unit (AU, multiplication of the absorbance with its dilution ratio for a certain sample). In order to compare with the data of intracellular *Monascus* pigment concentration, the concentration of extracellular *Monascus* pigments was also normalized to initial biomass concentration of 10 g lipid-free DCW per liter. In the case of cultivation in peanut oil–water two-phase system, intracellular *Monascus* pigments were exported near completely into the peanut oil phase. The peanut oil phase (0.9 g, corresponding to 1 ml) was washed with ethanol aqueous solution (70%, V/V, pH 2) until nearly no pigment was observed in the oil phase. Pigment concentration in the ethanol aqueous solution was determined, which was also normalized to the volume of water phase with initial biomass concentration of 10 g lipid-free DCW per liter.

In addition, supernatant (1 ml) of the nonionic surfactant micelle aqueous solution or water phase (1 ml) of peanut oil–water two-phase system was also diluted directly with water to determine the residual glucose concentration. The residual glucose concentration was determined by the standard 3,5-dinitrosalicylic acid method (DNS).

## Results

### Effect of cultivation condition on mycelia physiology

**Fig. 1:**
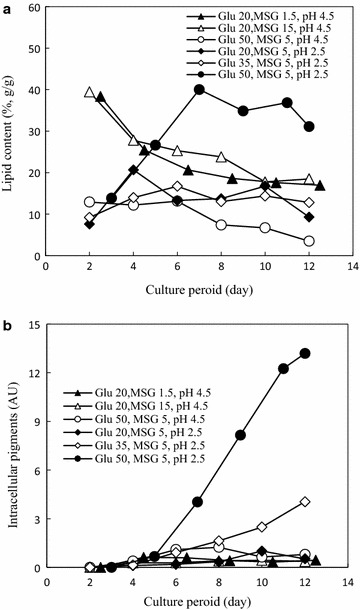
Effect of cultivation medium on microbial physiology. **a** Lipid content, defined as the lipid weight per 100 g lipid-free DCW; **b** intracellular *Monascus* pigments, normalized to biomass concentration of 10 g lipid-free DCW per liter. The data are average of triplicates. For clear vision, no *error bar* was represented

The cultivation media for extractive fermentation was divided into three groups (Table 1), i.e., different MSG concentration between entry 1 and entry 2, different pH between entry 3 and entry 4, and different glucose concentration between entry 5 and entry 6. The effect of cultivation medium as well as culture period on mycelia physiology, lipid content in mycelia (Fig. 1a) and intracellular *Monascus* pigment concentration (Fig. 1b), was examined. In most cases, the lipid content was kept below 20%. However, high lipid content was maintained at the early stage in the case of pH 4.5 and glucose 20 g/l (entry 1 and entry 2) or at the later stage in the case of pH 2.5, MSG 5 g/l, and glucose 50 g/l (entry 4) (Fig. 1a). On the other hand, the concentration of intracellular *Monascus* pigments was very low, as the presence of Triton X-100 in the media had exported the intracellular *Monascus* pigments into its extracellular broth (Hu et al. 2012). In the case of low pH and relatively high glucose concentration (entry 5 and entry 4), a relatively higher intracellular *Monascus* pigment concentration was observed at the later stage (Fig. 1b).

### Bioactivity of whole cell biocatalyst

**Fig. 2:**
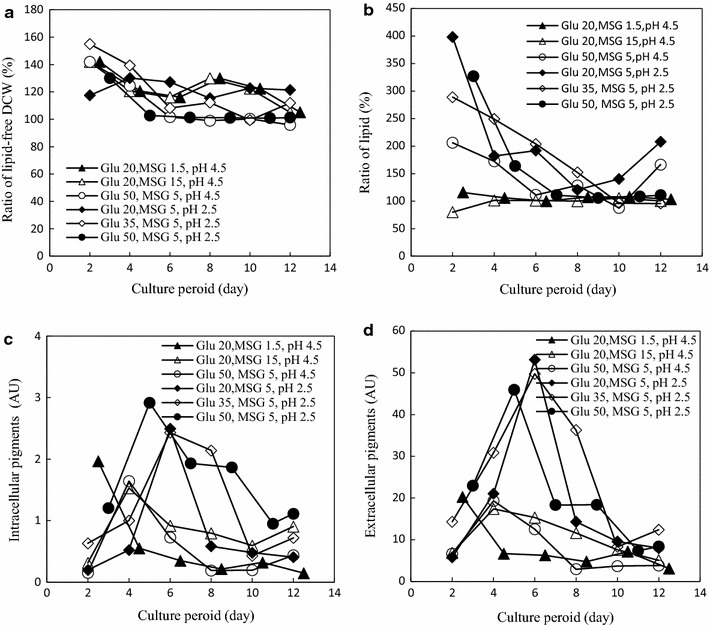
Cell suspension culture with mycelia collected from different cultivation conditions. **a** Change of biomass, defined as the ratio of lipid-free DCW after and before determination of mycelia bioactivity; **b** change of lipid content, defined as the ratio of lipid content after and before determination of mycelia bioactivity; **c** intracellular *Monascus* pigments concentration, normalized to biomass concentration of 10 g lipid-free DCW per liter; **d** extracellular *Monascus* pigments concentration, normalized to biomass concentration of 10 g lipid-free DCW per liter. The data are average of triplicates. For clear vision, no *error bar* was represented

The bioactivity of mycelia was determined by cell suspension culture using mycelia as whole cell biocatalyst. Cell suspension culture under the depletion of a compulsory nutrient element (MSG) condition, biomass (lipid-free DCW) increase was very limited. When mycelia were collected at the later stage of culture period, the increase of lipid-free DCW was no more than 20% (Fig. 2a). This result is consistent with the mycelia maintaining resting cell state under the depletion of a compulsory nutrient element condition (Willrodt et al. 2016). On the contrary, lipid content increased during the cell suspension culture (Fig. 2b). In comparison with the initial lipid content in the mycelia (Fig. 1b), it was found that high initial lipid content in the mycelia (such as the early stage of entry 1 and entry 2 as well as the later stage of entry 4) maintained nearly no increase of lipid content. On the other hand, mycelia with low initial lipid content, such as the early stage of culture period, lipid content increased very remarkable. In other words, relatively high lipid content in mycelia was achieved during cell suspension culture under the depletion of MSG condition.

Furthermore, orange *Monascus* pigments were also produced during cell suspension culture. The extracellular *Monascus* pigment concentration as well as the intracellular one was normalized to initial biomass concentration of 10 g lipid-free DCW per liter. In most cases, the concentration of intracellular *Monascus* pigments before (Fig. 1b) and after (Fig. 2c) cell suspension culture was neglectable. Thus, the concentration of extracellular *Monascus* pigments could be used to index the bioactivity of mycelia as whole cell biocatalyst for production of orange *Monascus* pigments (Fig. 2d). It was found that the accumulation of orange *Monascus* pigments depended not only on the cultivation media but also on cultivation period. The influence of MSG concentration on production of extracellular *Monascus* pigments was very limited (entry 1 and entry 2). However, the effect of pH on concentration of extracellular *Monascus* pigments was remarkable that low pH 2.5 led to high concentration of extracellular *Monascus* pigments (entry 3 and entry 4). The influence of pH on concentration of extracellular *Monascus* pigments was further confirmed under the relatively lower glucose concentration condition (entry 5 and entry 6). Thus, mycelia collected from cultivation condition with very low pH 2.5 (entry 4, 5, and 6) and at a middle culture period (approximately the 6th day) exhibited high bioactivity for production of high concentration of *Monascus* pigments. It should be pointed out that submerged culture with high glucose concentration 50 g/l and low pH 2.5 led to relatively higher intracellular *Monascus* pigment concentration at the later culture period (the 11th and 12th day of entry 4 in Fig. 1b). However, the corresponding extracellular *Monascus* pigment concentration was low after cell suspension culture of those mycelia as whole cell biocatalyst (Fig. 2d).

### *Monascus* pigment degradation/inhibition

Submerged culture was carried out in a medium with glucose 60 g/l, MSG 5 g/l, and pH 2.5 to the 10th day. Then mycelia were collected to further confirm the influence of intracellular *Monascus* pigments on mycelia bioactivity for production of orange *Monascus* pigments. Some of the mycelia were subjected to releasing their intracellular *Monascus* pigments by being washed with Triton X-100 (70 g/l) micelle aqueous solution. Both washed mycelia and no washed mycelia were used as whole cell biocatalyst for cell suspension culture for 42 h (Table 2). The lipid content of washed mycelia were almost unaffected while most of intracellular *Monascus* pigments were exported. After cell suspension culture, washed mycelia consumed glucose more rapidly. Defining accumulation of *Monascus* pigments as the sum of both intracellular *Monascus* pigments and extracellular ones after cell suspension culture and subtracting the initial intracellular *Monascus* pigments before cell suspension culture, relatively higher concentration of extracellular orange *Monascus* pigments confirmed the bioactivity of washed mycelia. On the contrary, a negative accumulation of *Monascus* pigments was observed during the cell suspension culture of no washed mycelia. The result indicated the pigment degradation rate under the experimental condition was faster than the pigment biosynthesis rate by using no washed mycelia as whole cell biocatalyst. No doubt, it is also possible that the relatively higher concentration of intracellular *Monascus* pigments has an inhibitory effect on bioactivity of no washed mycelia for production of orange *Monascus* pigments.

**Table 2:** Cell suspension culture using before and after washed mycelia as whole cell biocatalyst

	No washed mycelia	Washed mycelia
Initial loading mycelia		
Lipid-free mycelia (g/l)	13.63 (±0.02)	10.62 (±0.03)
Lipid content (%)	27.69 (±0.64)	26.88 (±0.55)
Intracellular *Monascus* pigments (AU)	31.96 (±0.51)	1.32 (±0.07)
After cell suspension culture		
Residual glucose (g/l)	43.41 (±0.05)	35.33 (±1.27)
Intracellular *Monascus* pigments (AU)	2.52 (±0.11)	0.63 (±0.07)
Extracellular *Monascus* pigments (AU)	18.01 (±0.21)	10.08 (±0.75)
Accumulation of *Monascus* pigments (AU)	−8.38 (±0.07)	8.84 (±0.71)

### Time course of cell suspension culture

**Fig. 3:**
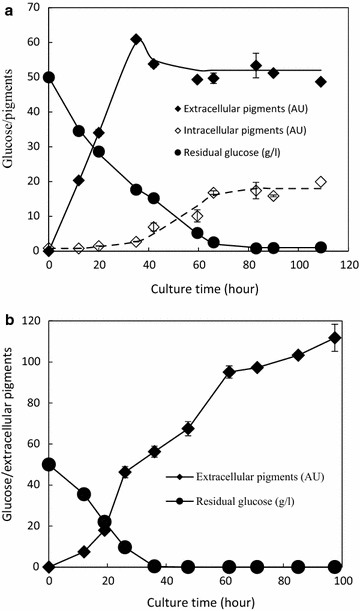
Time course of cell suspension culture. **a** In nonionic surfactant micelle aqueous solution with Triton X-100 70 g. Initial mycelia loading was 10 g lipid-free DCW per liter of the basic medium; **b** in peanut oil–water two-phase system with volume ratio of oil to water was 1:1. Initial mycelia loading was 12 g lipid-free DCW per liter of the basic medium. Mycelia were collected from extractive fermentation with glucose 35 g/l, MSG 5 g/l, and pH 2.5 (entry 5 in Table 1) at the 6th day

The time course of cell suspension culture using mycelia as whole cell biocatalyst in a nonionic surfactant micelle aqueous solution was further examined (Fig. 3a). The nonionic surfactant micelle aqueous solution had exported the intracellular *Monascus* pigments into its extracellular broth and then the *Monascus* pigments were solubilized in the micelles. At early stage (from beginning to the 40th h), glucose concentration decreased rapidly. The corresponding *Monascus* pigments were solubilized in micelles and the concentration of extracellular *Monascus* pigments increased linearly. Then (from the 40th to 66th h), the increase of extracellular *Monascus* pigment concentration stagnated while the concentration of intracellular *Monascus* pigments increased slowly with the consumption of glucose. Finally, glucose was degraded completely and both intracellular *Monascus* pigment concentration and extracellular one maintained stable.

Replacing nonionic surfactant with peanut oil as extractant, intracellular *Monascus* pigments were exported near completely into the peanut oil phase, while both intracellular *Monascus* pigment concentration and pigment concentration in the water phase were neglectable in the whole cultivation process (data not shown). Residual glucose concentration and the concentration of extracellular *Monascus* pigments were presented (Fig. 3b). At the beginning stage, glucose was consumed and extracellular *Monascus* pigments were produced. However, even without glucose consumption, the increase of pigment concentration was still observed with the further prolonged cell suspension culture time. Interestingly, a very high concentration of extracellular *Monascus* pigments (approximately 110 AU at 470 nm) was achieved while the corresponding intracellular *Monascus* pigments maintained neglectable.

## Discussion

The cultivation condition affects the microbial physiology and then the bioactivity of mycelia as whole cell biocatalyst (Chen et al. 2011; Olaofe et al. 2013; Ramesh et al. 2016). In the present work, mycelia were collected after submerged culture under different conditions. The bioactivity of using those mycelia as whole cell biocatalyst was determined by cell suspension culture in nitrogen (MSG)-free culture medium. The mycelia exhibited limited biomass (lipid-free DCW) growth (Fig. 2a), which indicated that mycelia were kept in resting cell state during cell suspension culture. The resting cells as whole cell biocatalyst showed bioactivity, such as production of lipids (Fig. 2b), biosynthesis of orange *Monascus* pigments (Fig. 2c, d) as well as consumption of glucose (Fig. 3a). The bioactivity for production of orange *Monascus* pigments was found to be related to the culture period. It consists with the fact that whole cell biocatalyst is usually harvested at the late logarithm phase during growing cell submerged culture (Oremland et al. 1999). It was also observed that low pH cultivation condition was a key factor for mycelia to exhibit high bioactivity for production of orange *Monascus* pigments (Fig. 2d). In our previous work, accumulation of intracellular orange *Monascus* pigments (Kang et al. 2013), inhibition of citrinin production (Kang et al. 2014), and high lipid content in biomass (Wang et al. 2015) at low pH are reported. It is attributed to the fact that nitrogen source is assimilated by microorganism via the way of controlled release of extracellular nitrogen source and maintenance of low intracellular nitrogen concentration under low pH condition (Kang et al. 2013). It is even reported that enzyme activity is regulated by nutritional requirements and is nitrogen-dependent (Hebert et al. 2000). However, no correlation between microbial physiology (lipid content or intracellular *Monascus* pigments as shown in Fig. 1) and bioactivity for production of orange *Monascus* pigments (Fig. 2d) was observed.

The bioactivity of mycelia as whole cell biocatalyst was very sensitive to high concentration of intracellular *Monascus* pigments (Table 2). This fact should be attributed to the instability of orange *Monascus* pigments under the experimental condition. At the same time, the inhibitory effect of product on the bioactivity of whole cell biocatalyst should also not be excluded. Bioactivity is an intrinsic character of mycelia as whole cell biocatalyst. However, it is usually underestimated or even neglected due to the measurement under an unfavorable experimental condition (Winter et al. 2014). Traditionally, biosynthesis of *Monascus* pigments is regarded as cell-growth dependence and production of *Monascus* pigments is usually carried out by microbial fermentation with growing cells (Chen et al. 2015). Benefiting from extractive fermentation in a nonionic surfactant micelle aqueous solution, intracellular *Monascus* pigments are exported out of cell interior and the extracellular *Monascus* pigments are solubilized in the nonionic surfactant micelles (Hu et al. 2012). And then, enzymatic kinetic study could be carried out by keeping biocatalyst in an environment with low product concentration (Fig. 2d; Table 2). Thus, production of orange *Monascus* pigments by mycelia as whole cell biocatalyst has also been recognized (Wang et al. 2016; Hu et al. 2016).

Segregation of product from biocatalyst for elimination of product degradation/inhibition is not only necessary for study of enzymatic kinetics but also beneficial to industrial application of mycelia as whole cell biocatalyst. Time course of cell suspension culture in a nonionic surfactant micelle aqueous solution indicated that product degradation as well as inhibition was eliminated at early stage (before reaching the extraction capacity of nonionic surfactant micelles) and then intracellular *Monascus* pigments were accumulated at the later stage (after reaching the extraction capacity of nonionic surfactant micelles, which is similar to traditional fermentation in an aqueous solution) (Fig. 3a). Thus, extraction capacity of nonionic surfactant micelles was related to elimination of product degradation/inhibition and then related to product accumulation, which had been further confirmed by replacing nonionic surfactant with peanut oil as extractant (Fig. 3b). Accumulation of high concentration of extracellular *Monascus* pigments was realized due to the plant oil phase with high extractive capacity. Furthermore, instable/toxic extracellular product can also be converted into relatively stable/nontoxic compound by enzymatic reaction (Willrodt et al. 2015) or non-enzymatic reaction (Xiong et al. 2015; Domaille et al. 2016; Wallace and Balskus 2016), which may be a more efficient strategy to make whole cell biocatalyst play its potential.

In conclusion, collection of optimized mycelia as robust biocatalyst for production of orange *Monascus* pigments has been achieved by submerged culture in a nonionic surfactant micelle aqueous solution at low pH 2.5. The bioactivity of mycelia as whole cell biocatalyst is sensitive to high product concentration, which is attributed to the product degradation/inhibition. Segregation of product from biocatalyst by cell suspension culture in a nonionic surfactant micelle aqueous solution or peanut oil–water two-phase system is not only necessary for studying the enzymatic kinetics but also beneficial to industrial application of mycelia as whole cell biocatalyst.

## Abbreviations

MSG: monosodium glutamate
DCW: dry cell weight
